# Untargeted LC-MS metabolomics reveals the metabolic responses in olive flounder subjected to hirame rhabdovirus infection

**DOI:** 10.3389/fimmu.2023.1148740

**Published:** 2023-08-28

**Authors:** Bingyu Gu, Fenghuang Pan, Hongxiang Wang, Zhiyi Zou, Junya Song, Jing Xing, Xiaoqian Tang, Yuanchao Zhan

**Affiliations:** ^1^ College of Marine Life Science, Ocean University of China, Qingdao, China; ^2^ Laboratory of Pathology and Immunology of Aquatic Animals, Laboratory of Pathology and Immunology of Aquatic Animals, Key Laboratory of Mariculture, Ministry of Education (KLMME), Fisheries College, Ocean University of China, Qingdao, China; ^3^ Haide College, Ocean University of China, Qingdao, China

**Keywords:** HIRRV, olive flounder, metabolomic, immune response, viral infection

## Abstract

Hirame novirhabdovirus (HIRRV), which mainly infects the olive flounder (*Paralichthys olivaceus*), is considered to be one of the most serious viral pathogens threatening the global fish culture industry. However, little is known about the mechanism of host-pathogen interactions at the metabolomic level. In this study, in order to explore the metabolic response of olive flounder to HIRRV infection, liquid chromatography mass spectrometry (LC-MS) was used to detect the changes of endogenous compounds of the olive flounder after HIRRV infection. A total of 954 unique masses were obtained, including 495 metabolites and 459 lipids. Among them, 7 and 173 qualified differential metabolites were identified at 2 days and 7 days post-infection, respectively. Distinct metabolic profiles were observed along with viral infection. At the early stage of infection, only a few metabolites were perturbed. Among them, the level of inosine and carnosine were increased and the potential antiviral ability of these two metabolites was further confirmed by exogenous addition experiment. At the late stage of HIRRV infection, the metabolic profiles changed remarkably. The changes in amino acids and nucleotides especially the 7-methylguanine also accelerated the amplification of viral particles. And the down-regulation of glutathione (GSH) implied an elevated level of ROS (reactive oxygen species) that attenuated the immune system of flounders. HIRRV also induced the accumulation of purine and reduction of pyrimidine, and elevated LPC and LPE levels. The unbalanced purine/pyrimidine and altered lipid profile may be beneficial for the replication and infection of HIRRV at the late stage of infection. These findings provide new insights into the pathogenic mechanism of HIRRV infection in olive flounder.

## 1 Introduction

Olive flounder (*Paralichthys olivaceus*), as an important marine fish, is widely distributed in the coast of China, Japan and Korea (1). Since the 1990s, it has become a major aquaculture species in Asia, greatly promoting the local aquaculture economy (2, 3). Hirame novirhabdovirus (HIRRV), a member of *Novirhabdovirus* within Rhabdoviridae, was first isolated from olive flounders in the 1980s and later spread to Korea, China, and even Poland (4, 5). HIRRV has a broad host range, including stone flounder (*Kareius bicoloratus*), sea bass (*Lateolabrax maculatus*), and black seabream (*Acanthopagrus schlegeli*) (6). This viral infection leads to a severer hemorrhage in the muscle, mesentery and subcutaneous tissues, eventually causes the massive death of the infected fish (5). Therefore, HIRRV is considered as one of the most serious viral pathogens, threatening the safety of current fishery industry (7).

Several studies investigated the potential defense mechanism and preventive strategies of HIRRV infection in olive flounder. A transcriptomic study has revealed that HIRRV can effectively activate the inflammatory and immune-related pathways, particularly RIG-I-like receptor signaling pathway in defense against HIRRV infection (8). Further study found that HIRRV-G protein could induce mucosal and humoral immunity of olive flounder, which laid a foundation for oral vaccine development. Both DNA vaccines and deactivated or recombinant vaccines have also been developed to control pathogen infection. Unfortunately, none of these vaccines is commercially available yet (9–11). There is an increasing necessity to have an in-depth understanding of the mechanism of HIRRV infection and the antiviral response of flounder, which will be a prerequisite for disease control.

Metabolomics, complementary to genomics, transcriptomics and proteomics, is an omics method used to describe the profile of small organic molecules and the associated metabolic processes in cells (12). Due to the rapid, unbiased and quantitative information that metabolomics provides, it has been widely applied in the field of aquaculture. Particularly, metabolomics reveals the signature biomarkers and key metabolic pathways associated with bacterial and viral infection, providing a new method for early diagnosis and epidemic prevention (13). Metabolic responses to *Edwardsiella* infection have been investigated in various fish, such as tilapia and crucian carp (14, 15). In terms of viral infection, the metabolic changes of flounders subjected to hemorrhagic septicemia virus (VHSV) infection were already described. The metabolome of the article profiling revealed several key protective metabolites against VHSV infection, including the activation of the immune system and protein synthesis, and suppression of ATP synthesis and the antioxidant system (16). The metabolic profiles of rock bream in response to rock bream iridovirus (RBIV) infection were systematically studied, revealing an elaborated mechanism of the host-pathogen interaction (17). These studies unfolded key metabolites and pathways against infectious diseases, which provided a foundational understanding of infection mechanisms and improved protection strategies of fish against viral diseases.

In this study, we investigated the metabolic mechanism of HIRRV infection in olive flounder using liquid chromatography-mass spectrometry (LC-MS). The differentially expressed metabolites subjected to HIRRV infection were unfolded along with viral infection. We focused on the effects of HIRRV infection on the metabolism of olive flounders. The linkage between metabolite changes with the immunological response and viral proliferation was also elucidated. Our study provides a better understanding of the mechanism of HIRRV infection and the defense system of olive flounder against viral infection, which allows for a proposal of a potential therapeutic intervention in disease management of aquaculture production.

## 2 Materials and methods

### 2.1 Virus infection and sampling

Juvenile flounders with a body length of 23 ± 3 cm and body weight of 120 ± 20 g were collected from a marine fish farm in Rizhao, China. The fish were grown at 10°C in the lab under diel light cycles. They were fed with food twice per day (50 g/time), and the 1/3 of water was changed every other day.

The HIRRV CNPo2015 strain was obtained as previously mentioned (5). Briefly, the virus was isolated from infected *Paralichthys olivaceus*, and further amplified in epithelioma papulosum cyprini cell lines. After a large range of cytopathic effects appeared, the HIRRV was collected by centrifugation. The titer of the HIRRV was measured by the method described by Reed and Muench (18) and adjusted to 1.0 ×10^7.8^ TCID_50_/mL. Fish were intraperitoneally injected with 100 μL HIRRV. Before sampling, 100 ng/mL MS-222 was administered as anesthesia. The peripheral blood and the spleen of flounders were collected immediately (0 day), at 2 and 7 days post-infection (dpi) for metabolic analysis and pathogen detection (n=10). Blood was collected from the tail vein and stored overnight at 4°C to allow clotting, and serum was obtained by centrifugation at 8000×g for 15 minutes. The collected serum and spleen were stored at -80°C for further analysis.

### 2.2 Quantification of HIRRV

Trizol method was used to extract total RNA from the spleen of HIRRV-infected flounders, and cDNA was obtained by reverse transcription. NanoDrop 8000 (ThermoFisher, USA) was used to determine the concentration of cDNA, and adjusted to 1000 ng/μL. HIRRV G gene was used as the detection target to measure the concentration of HIRRV by qPCR (See **Table S1** for primers) as previously described (19).

### 2.3 Sample preparation and extraction

To extract hydrophilic compounds, the serum sample was defrosted on ice, and 3 volumes of ice-cold methanol were added to 1 volume of serum. The mixture was whisked for 3 minutes and then centrifuged at 12,000 rpm for 10 minutes at 4°C. Then the supernatant was collected and centrifuged at 12,000 rpm at 4°C for 5 minutes. The final supernatant was collected and used for LC-MS/MS analysis.

For hydrophobic compounds extraction, the sample was thawed on ice and then centrifuged with 3000 rpm at 4°C for 5 minutes. 50 μL of the sample with 1 mL extraction solution (include methanol, MTBE and internal standard mixture) added was homogenized and the mixture vortexed for 2 minutes. 500 μL water was added and mixed for another 1 minute. The sample was then centrifuged with 12,000 rpm at 4°C for 10 minutes. The supernatant was collected and concentrated with a vacuum concentrator at 4°C. The sample was resuspended using 100 μL mobile phase B (acetonitrile with 0.04% formic acid), then stored in -80°C for later LC-MS/MS analysis.

### 2.4 UPLC conditions

For hydrophilic substances, Shim-pack UFLC SHIMADZU CBM A system, a Waters ACQUITY UPLC HSS T3 C18 (1.8 μm, 100 × 2.1 mm) column at a flow rate of 0.4 ml/minutes were used. The injection volume was 2 μL and the temperature was 40°C. Solvent A for the hydrophilic substance mobile phase is ultra-pure water containing 0.1% formic acid, solvent B is acetonitrile containing 0.1% formic acid. The hydrophilic substances were eluted at 0 minute (water/acetonitrile = 95:5 V/V), 11.0 minutes (water/acetonitrile = 10:90 V/V), 12.0 minutes (water/acetonitrile = 10: 90 V/V), 12.1 minutes (water/acetonitrile = 95:5 V/V) and 14.0 minutes (water/acetonitrile = 95:5 V/V).

For hydrophobic substances, the same system, column, temperature and the injection volume were utilized. The hydrophobic mobile solvent A is ultra-pure water containing 0.04% formic acid, and the solvent B is acetonitrile containing 0.04% formic acid. Elution was carried out with different gradient at 0 minute (water/acetonitrile = 95:5 V/V), 11.0 minutes (water/acetonitrile =5:95 V/V), 12.0 minutes (water/acetonitrile = 5:95 V/V), 12.1 minutes (water/acetonitrile = 95:5 V/V) and 14.0 minutes (water/acetonitrile = 95:5 V/V).

### 2.5 ESI-Q TRAP-MS/MS condition

The serum metabolome and lipid profiles of hydrophilic and hydrophobic materials were analyzed using AB Sciex Qtrap 6500+ equipped with an ESI Turbo Ion-Spray interface, operating in positive and negative ion synchronous mode and controlled by Analyst software (1.6.3).

The ESI source operation parameters were as follow: ion spray voltage was set at 5500 V (positive) and -4500 V (negative); temperature was 550°C; the pressures for ion source gas I (GSI), ion source gas II (GSII), and gas curtain gas (CUR) were set at 55 psi, 60 psi, and 25 psi, respectively. The collision-activated ionization (CAI) parameter was set to high. In the triple quadrupole (QQQ), each ion was scanned for optimized detection using decluttering potential (DP) and collision energy (CE). QQQ was scanned by multiple reaction monitoring (MRM). Mass calibration was performed using 100 μM and 10 μM polypropylene glycol solutions for online Ion Trap (LIT) and QQQ modes, respectively (13).

### 2.5 Metabolomic data acquisition

To obtain high-quality mass spectrometry data, quality control samples (QC) were used throughout the experiment (20–22). MRM model was used to quantify metabolites. After obtaining the offline data of samples, MultiQuant software (3.0.3) was used to integrate the extracted chromatographic peak areas of all metabolites. After correction, the relative abundance of metabolites and the integrated data of all chromatographic peak areas was obtained.

### 2.6 Data processing and analysis

Orthogonal partial least squares discriminant analysis (OPLS-DA) was performed to reveal the global metabolic changes among groups. The data was logarithmically transformed before applying OPLS-DA. Two parameters, model fit (R^2^) and prediction quality (Q^2^) were used to evaluate the validity of the model. A permutation test (200 times) was executed to further verify the quality of the OPLS-DA model.

Differential metabolites between groups were determined by student’s t-test based on the following criteria: variable importance in projection (VIP) ≥ 1, fold change (FC) ≥ 2 or ≤ 0.5, and P < 0.05. To minimize possible false positive results, the Benjamini-Hochberg procedure was conducted to correct the P-value. The KEGG database was used to further screen metabolites that could be matched to metabolic pathways. GraphPad 9 and R (4.1.2) (ggplot2) were used for data visualization.

### 2.7 Cellular infection assay

To evaluate the effect of differentially expressed metabolites on HIRRV-infected flounder, specific metabolites, including inosine, carnosine and glutamine, glycine, and 7-methylguanine, were selected for experimental verification after exposure to HINAE cells after virus infection. Specifically, HINAE cells were cultured in 6-well plates at 20°C in Leibovitz’s -15 medium (Gibco, USA) supplemented with 10% fetal bovine serum (Gibco, USA), 100 IU/mL penicillin and 100 μg/mL streptomycin. For experimental group, the cells were infected with HIRRV at a multiplicity of infection (MOI) of one for 1 hour. Subsequently, inosine, carnosine glutamine, glycine, 7-methylguanine were diluted with PBS, and added to HINAE cell at a final concentration of 0.15 mM, 0.1 mM, 4 mM, 6.8 mM, and 0.1 mM, respectively. After 48 h, total RNA of cells was extracted to detect copy number of HIRRV by RT-qPCR. The cytotoxicity of these metabolites were determined by Cell Counting Kit (CCK-8,Vazyme) 24 hours after supplement.

In addition, to validate our metabolomics results, we also measured the expression of 5’-methylthioadenosine (MTA), GSH, and cystine metabolism-related genes in the infected group of the late metabolite group. All experiments were performed in triplicate, and primers used in this study was shown in **Table S1**.

### 2.8 Enzyme-linked immunosorbent assay(ELISA)

To verify the finding obtained through LC-MS/MS, the level of flounder inosine, carnosine and LPC were detected in both HIRRV infected and health HINAE cells using corresponding ELISA kits (Ruifan, Fantai). The cell culture and virus infection procedures were performed as previously described. The cell supernatant was collected at 12, 24 and 48 hours after infection and used for subsequential ELISA measurement.

## 3 Result

### 3.1 Detection of HIRRV infection

**Figure 1:**
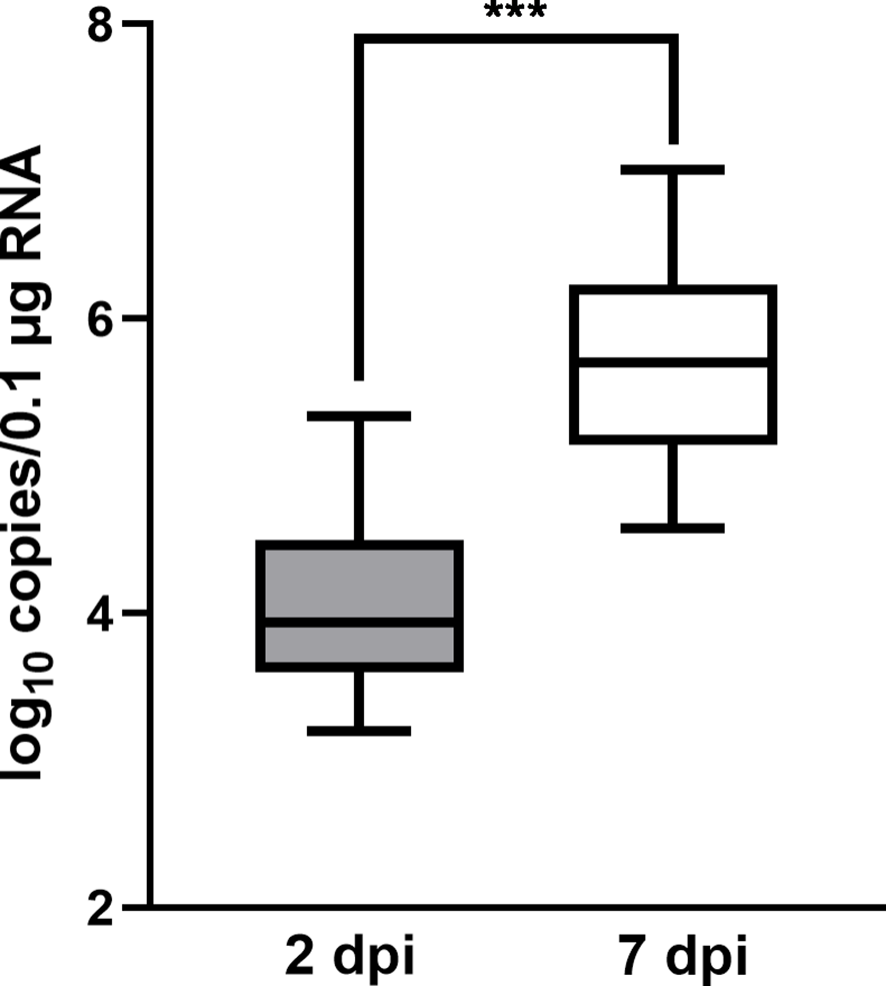
The quantity of HIRRV in the spleen of founders measured by RT-qPCR (***, *p* < 0.001).

To validate the infection of flounders by HIRRV, the copy numbers of HIRRV were quantified in the spleen of flounders along with viral infection. The mean copy numbers of HIRRV were 3.31x10^4^ and 1.73x10^6^ copies/0.1 μg RNA at 2 dpi and 7 dpi, respectively (**Figure 1**). The pathogen load within flounders at 7 dpi was found to be more than 50 times higher than that at 2 dpi (P < 0.001). Meanwhile, the anatomical observation of flounder at the late stage of infection showed that there were obvious symptoms of infection in its viscera (**Figure S1**).The substantially increased HIRRV is consistent with picture observations of the observed severe symptoms and increased cumulative mortality rate along with viral infection observed previously (5).

### 3.2 HIRRV induced global metabolic changes of the flounder

A total of 954 unique masses were identified, among which 495 metabolites and 459 lipids were putatively annotated by hydrophilic interaction liquid chromatography (HILIC) and reversed-phase liquid chromatography (RPLC), respectively.

**Figure 2:**
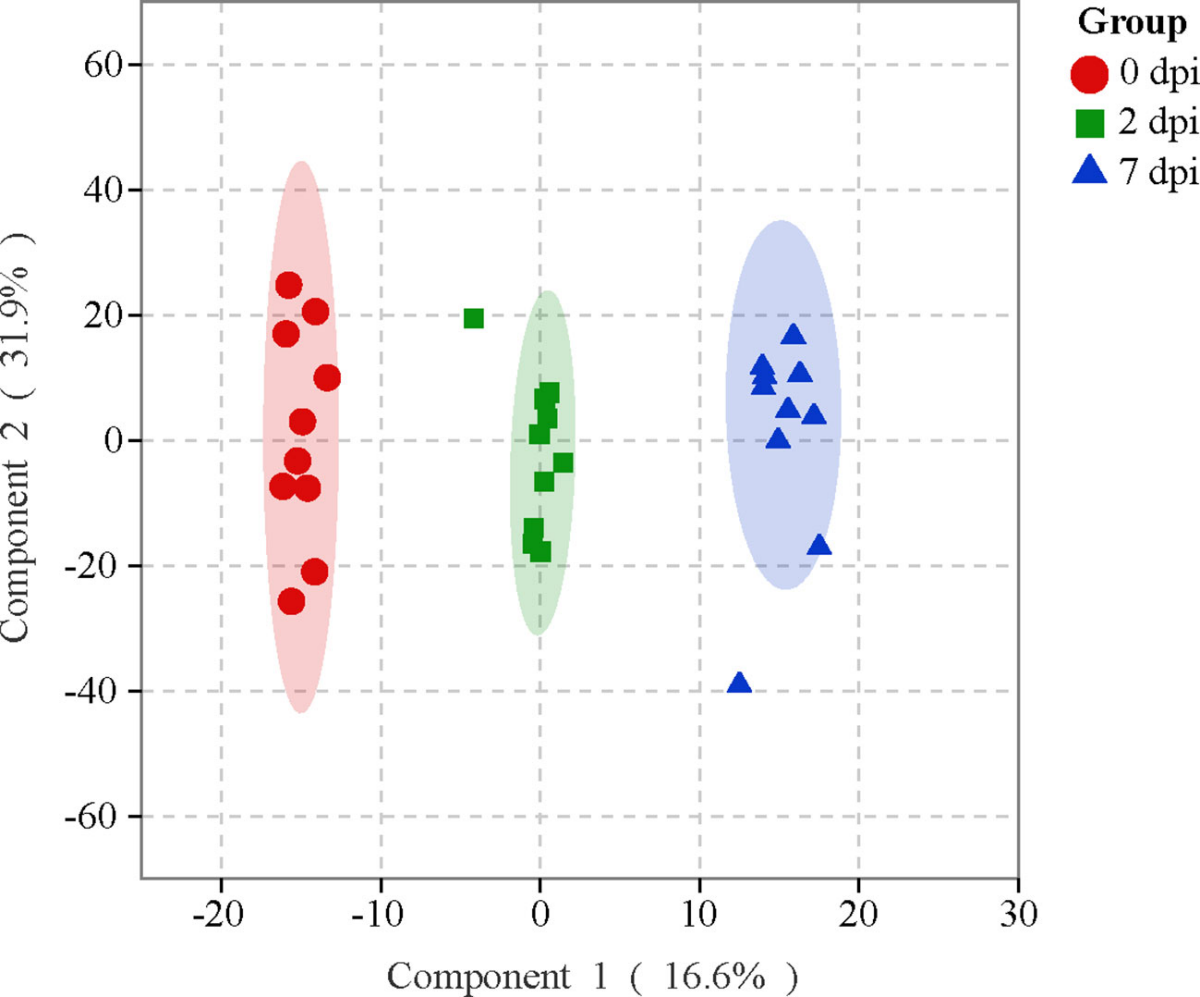
The OPLS-DA score plot reveals the overall metabolic profiles of flounders subjecting to HIRRV infection.

The OPLS-DA was applied to investigate the overall metabolic differences along the viral infection. Despite a few outliers, a clear separation was observed among the samples collected at 0, 2, and 7 dpi (**Figure 2**). A multiple correlation coefficient (R^2^) of 95.6% and a permutation test further indicate an optimal fitness and prediction performance of the OPLS-DA model (**Table S2**). Therefore, the HIRRV infection significantly perturbed the global metabolic profile of flounders.

### 3.3 A various categories of metabolites altered subject to viral infection

Differential metabolites among groups were filtered based on the criteria that fold change (FC) ≥ 2 or ≤ 0.5, VIP ≥ 1 and P < 0.05. After data processing, 7 and 173 metabolites were identified in the 2 and 7 dpi groups compared to the 0 dpi group. Almost all of these different metabolites (97%) were discovered from the late stage of viral infection (7 dpi). Two-thirds of metabolites were decreased in abundance when facing viral infection.

**Figure 3:**
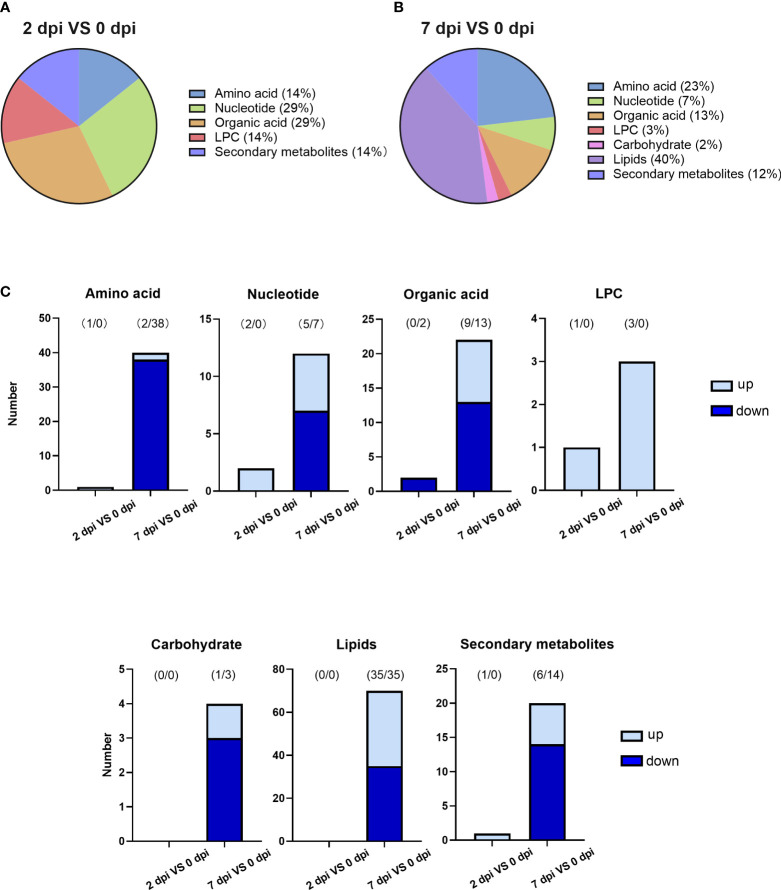
HIRRV infection disturbed various types of metabolites in flounders. **(A)** The categories of differential metabolites along the viral infection at the early stage of viral infection (2 dpi against 0 dpi). **(B)** The categories of differential metabolites at the late stage of viral infection (7 dpi against 0 dpi). **(C)** The number of changed metabolites within each category.

Only 7 metabolites were differentially expressed in the sample collected 2 days after infection (**Table S3**). However, they were widely dispersed into five different categories, including amino acids, nucleotide, organic acids, lysophosphatidylcholine (LPC), and secondary metabolites (**Figure 3A**). Five out of 7 metabolites were upregulated at 2 dpi (**Figure 3C**), while pimelic acid and suberic acid were the only two substances that were downregulated. These differently expressed metabolites could be potential indicators of early infection.

An upsurge in various metabolites was observed 7 days after the viral infection. A total number of 173 metabolites were significantly disturbed, which is more than 10 times higher than that of early infection (**Table S4**). New types of metabolites, lipids, and carbohydrates, were altered along with viral infection (**Figure 3B**). The lipids were significantly affected by viral infection at the late stage, occupying the largest proportion of disturbed metabolites (**Figure 3C**). Amino acids rank second at the late viral stage. 95% of amino acids decreased in abundance (**Figure 3C**). Lipids and amino acids strongly contribute to the metabolic differences caused by virus infection at the late stage.

### 3.4 Discovery and identification of differentially expressed metabolites

**Figure 4:**
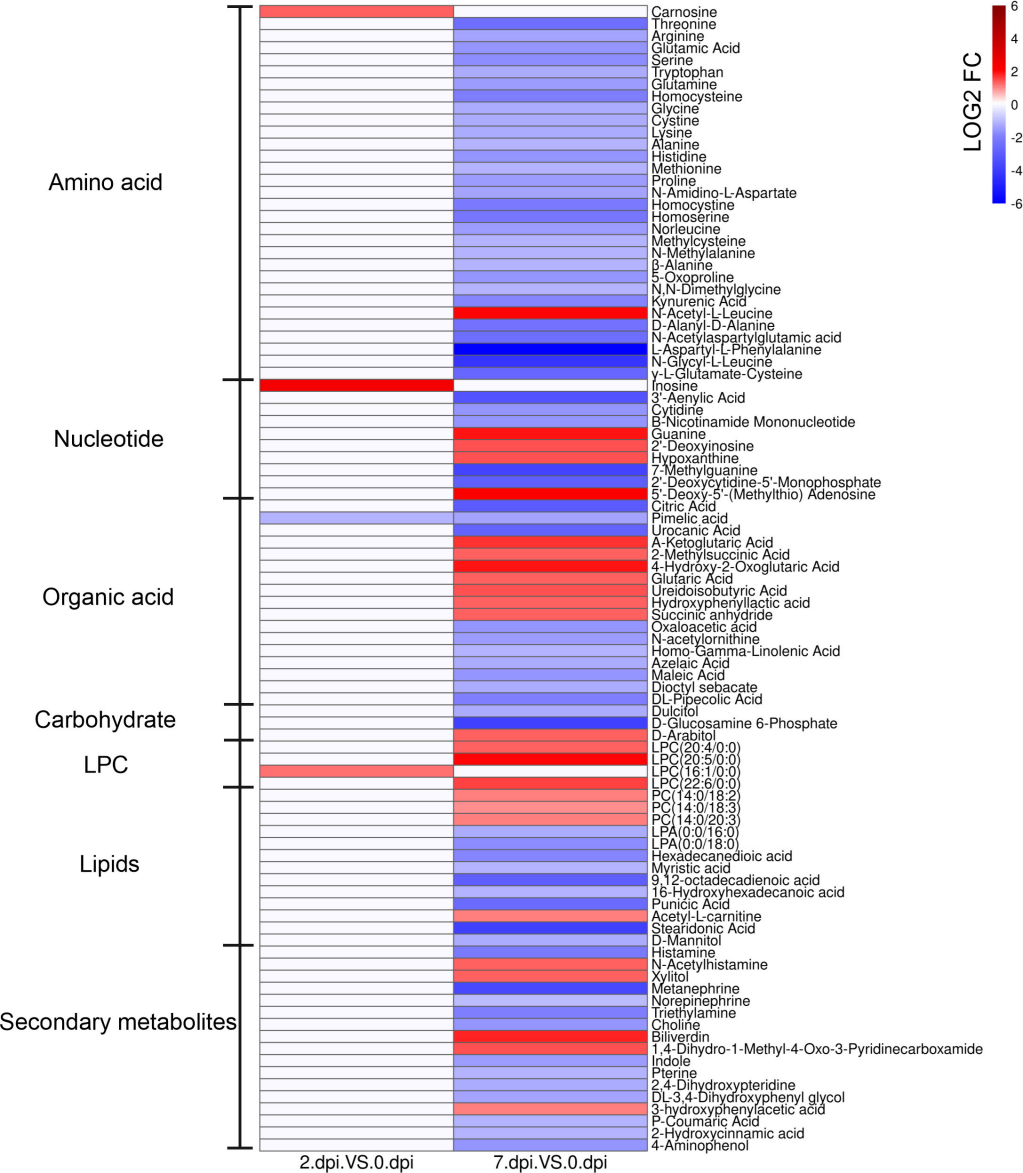
The heatmap of significantly different metabolites in serum of *Paralichthys olivaceus* after HIRRV infection. The abundance of metabolites was log-transformed.

To identify alterations in metabolism in response to HIRRV infection, metabolites that could be matched to pathways were selected based on the KEGG database. The annotated differential metabolite profiles in response to viral infection at 2 dpi and 7 dpi were displayed in the heatmap (**Figure 4**).

Two days after HIRRV infection, carnosine, inosine, and LPC (16:1/0:0) were substantially up-regulated, while the abundance of pimelic acid was reduced by more than 2 times (**Figure 4** and **Figure S2**).

At the late stage of viral infection, more metabolites were significantly altered. Almost all of the amino acids were down-regulated, including threonine, serine and alanine. Specifically, the expression of cystathionine gamma-lyase (CTH), which related to the synthesis of thiocysteine from cystine, was up-regulated after HIRRV infection (**Figure S3**). Not only monomeric amino acids, an overall decrease of dipeptides was also observed, including D-alanyl-D-alanine, N-amidino-L-aspartate, L-alanyl-L-lysine, etc. Among these dipeptides, it is worth mentioning that the abundance of L-aspartyl-L-phenylalanine, a precursor of many amino acids (23), and N- glycyl-L-leucine exhibited a remarkable decline by a factor of 113 and 19 times, respectively (**Figure 4** and **Figure S2**). Interestingly, in contrast to the overall downward trend, N-acetyl-L-leucine (NALL) became the only elevated annotated amino acid in the late-infection group. Our result also showed that the flounder adjusted the level of several amino acid derivatives subjected to the HIRRV infection. An up-regulations of MTA exhibited in the late stage. Consistently, an eight-fold increase in the expression of the spermine synthase (SMS) gene, which is responsible for producing of MTA from SAM, was observed during HIRRV infection in flounder HINAE cell. A considerable reduction in GSH levels was also noted, which aligns with the increased expression of glutathione S-transferase (GST) (**Figure S3**).

The abundance of many key molecules in the TCA cycle was also changed at the late stage of viral infection, including 2-oxoglutaric acid, citrate, and oxaloacetic acid. Despite the overall decrease of amino acids and metabolites involved in the TCA cycle, a three-fold increase in 2-oxoglutaric acid was observed (**Figure S2**).

In terms of lipids, both lysophosphatidylcholine (LPC) and lysophosphatidylethanolamine (LPE) in the flounder were accumulated in the serum when subjected to HIRRV infection; all of which were unsaturated (**Figure 4**). In contrast, the abundances of polyunsaturated fatty acids (PUFA) were down-regulated with a maximum fold change of 13.9.

### 3.5 The effects of screened metabolite addition on HIRRV infection

**Figure 5:**
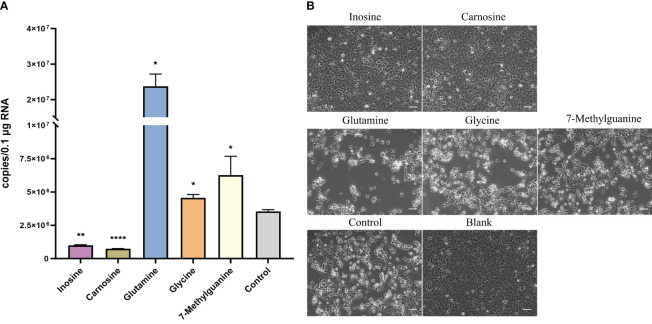
Effect of selected metabolites on viral proliferation after HIRRV infection at cellular level. **(A)** The number of HIRRV was detected by RT-qPCR 48hours after infection in the experimental groups supplemented with inosine, carnosine, glutamine, glycine and 7-methylguanine at the cellular level (*: p < 0.05, **: p < 0.01, ****: p < 0.0001). **(B)** Cytopathic effect (CPE) was observed in the experimental group with exogenous supplementation of inosine, carnosine, glutamine, glycine, and 7-methylguanine metabolites than in the control group. The blank indicates the no virus inoculation. Scale bars = 50 µm.

To investigate the effect of metabolites on HIRRV infection, several metabolites were selected and their effects were further investigated. Inosine and carnosine, which only increased at early stage of infection were supplemented after HIRRV infection. Compared with the control group, the HIRRV copy number was reduced by 3.6 and 4.1 times in these groups supplemented with inosine and carnosine, respectively (**Figure 5A** and **Figure S4**). In contract, three metabolites, glutamine, glycine, and 7-methylguanine, which significantly changed at late stage of HIRRV infection, could promote the HIRRV infection. The treatment groups exhibited a significant increase in HIRRV copy numbers compared to the control group (**Figure 5A** and **Figure S4**). Notably, the addition of glutamine induced a 4.7-fold increase in virus abundance.

Consistent with the viral abundance, treatment groups with glutamine, glycine, and 7-methylguanine, showed more severe cytopathic effect (CPE) consisting of round and granular cells, grape-like clusters, cell detachment and lysis two days after HIRRV infection compared with the control group (5). While adding inosine and carnosine led to a lesser degree of CPE (**Figure 5B**). No occurrence of CPE was observed in the blank group.

Overall, these findings suggest that inosine and carnosine are highly likely to help the host resist viral growth, while the other three metabolites may contribute to increased viral abundance and cytopathic effects.

### 3.6 The concentration of specific metabolites along with HIRRV infection by ELISA

**Figure 6:**
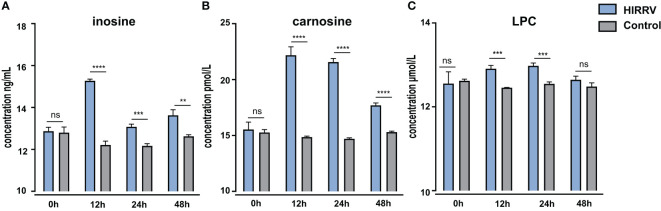
The level of inosine **(A)**, carnosine **(B)**, and LPC **(C)** in HINAE cells after HIRRV infection measured by ELISA. Asterisks (*) on the bar represent the statistically significant difference as compared to corresponding control group (ns : no significant, **: *p* < 0.01, ***: *p* < 0.001, ****: *p* < 0.0001).

To validate the observation obtained from LC-MS/MS, ELISA was employed to assess the levels of inosine, carnosine, and LPC in the supernatant of HINAE cells post HIRRV infection (**Figure 6**). In the control group, the abundance of inosine, carnosine and LPC remained relatively stable throughout the observation period. However, in the viral infection group, there was a significant surge in inosine levels at 12 hours post-infection, followed by a gradual decrease as the viral infection progressed. Regarding carnosine, the ELISA measurement showed a notable approximately 1.5-fold increase was observed at 12 hours post-viral infection, and this elevated level persisted until the 24-hour. The viral infection group exhibit approximately 1.5-fold increasing in carnosine in ELISA measurement. Furthermore, HIRRV infection had an impact on the content of LPC, leading to an increase in LPC levels. Together, these findings concurred well with the changes of metabolite level observed in metabolomic analysis.

## 4 Discussion

Viral infection was considered a major threat to aquaculture. In this study, we investigated a series of metabolic changes caused by HIRRV infection in olive flounder. Significant changes in metabolites were detected related to the immune system and antioxidant system of flounder, whereas, the altered nucleotide and lipid metabolisms might support replication and infection of HIRRV.

### 4.1 Inosine and carnosine, potential antiviral metabolites

During the initial phase of viral infection, there was a significant accumulation of inosine and carnosine at 2 dpi (**Figure S2**). This observation was further corroborated using ELISA, which provided similar trends, providing robust support for our metabolomic findings (**Figure 6**). The combined results from both metabolomic analysis and ELISA measurements confirmed that HIRRV infection indeed triggers substantial alterations in these two metabolites during the early stages of infection.

Supplementary experiments additionally demonstrated that the introduction of inosine and carnosine led to a significant reduction in HIRRV replication (**Figure 5A**), suggesting that these compounds might be beneficial for the host in resisting the extensive proliferation of HIRRV. Prior research has indicated a sharp increase in T-cells induced by HIRRV infection (24). Notably, inosine has been reported as an alternative carbon source for the proliferation and vitality of T-cells (25), while carnosine has been shown to enhance the metabolic fitness of activated T-cells (26). The elevation of these two metabolites potentially elucidates the improved functionality of T-cells during the early phase of HIRRV infection.

Consequently, the increase in inosine and carnosine during infection represents an essential strategy to impede the proliferation of HIRRV during the initial stages. Furthermore, considering their potential as feed supplements, inosine and carnosine could be considered to aid flounders in resisting HIRRV infection effectively.

### 4.2 Amino acids demanding by both T-cell and HIRRV

Amino acids have profound effects on both cells and pathogens (27). Our metabolic analyses have also identified changes in amino acid levels that might be involved in T-cell and HIRRV metabolism. Exogenous addition assays have further confirmed the critical role of amino acids in HIRRV infection.

At the late stage of infection, T-cells dropped rapidly (24). The majority of amino acids underwent a decrease in abundance at 7 dpi, which could contribute to the decrease of T-cells. Amino acids could promote T-cell function by producing biomass and energy, and reprogramming the cell metabolism (28). The decrease of serine, the major carbon donor of purine and thymidine, could fatally impair T-cell proliferation and function (**Figure S2**) (29). Glutamine also plays a vital role in T-cells proliferation, by providing energy through replenishing the TCA cycle (30, 31). The decreased glutamine and overall downregulated TCA cycle indicate the shortage of ATP for T-cells. In addition, MTA is a crucial intermediate involved in the methionine salvage pathway, also a product and regulator of the polyamine pathway. In our study, the abundance of MTA was considerably elevated by 4.5-fold at the late stage of viral infection (**Figure S3**). Several studies have demonstrated that the accumulation of MTA could suppress the proliferation, differentiation and function of T-cells by inhibiting Akt phosphorylation (32–34).

In addition, the deduction of various amino acids might utilize by HIRRV for its replication and proliferation. Our metabolome data showed that the intracellular level of glycine, glutamate and cysteine, were significantly reduced (**Figure S2**). Further supplementation experiment also revealed that addition of glycine and glutamine could significantly increase the replication of HIRRV (**Figure 5A**), suggesting that these amino acids probably were utilized for HIRRV production. Previous study showed that glycine plays a critical role in promoting Hepatitis C virus (HCV) RNA replication by promoting the establishment of functional replication organelles in cells (35). It has also been shown that glutamine deficiency can lead to a significant decrease in the particle number of vaccinia virus (VACV) (36), and glutamine is also essential for HIV and HCMV infection and replication (37, 38). Therefore, the modulation of amino acid during infection is an important strategy for HIRRV production.

Together, our results suggest that the reduction of essential amino acids has an impact on both the immune response of flounder and the replication of HIRRV. The depletion of key amino acids, in combination with the increased level of MTA, inhibits T-cell function and impairs the flounder immune system during the late stage of HIRRV infection. Additionally, the amino acids are redirected to viral particles, resulting in a significant increase in HIRRV abundance (**Figure 5A**).

### 4.3 Nucleotide metabolism and its methylation

**Figure 7:**
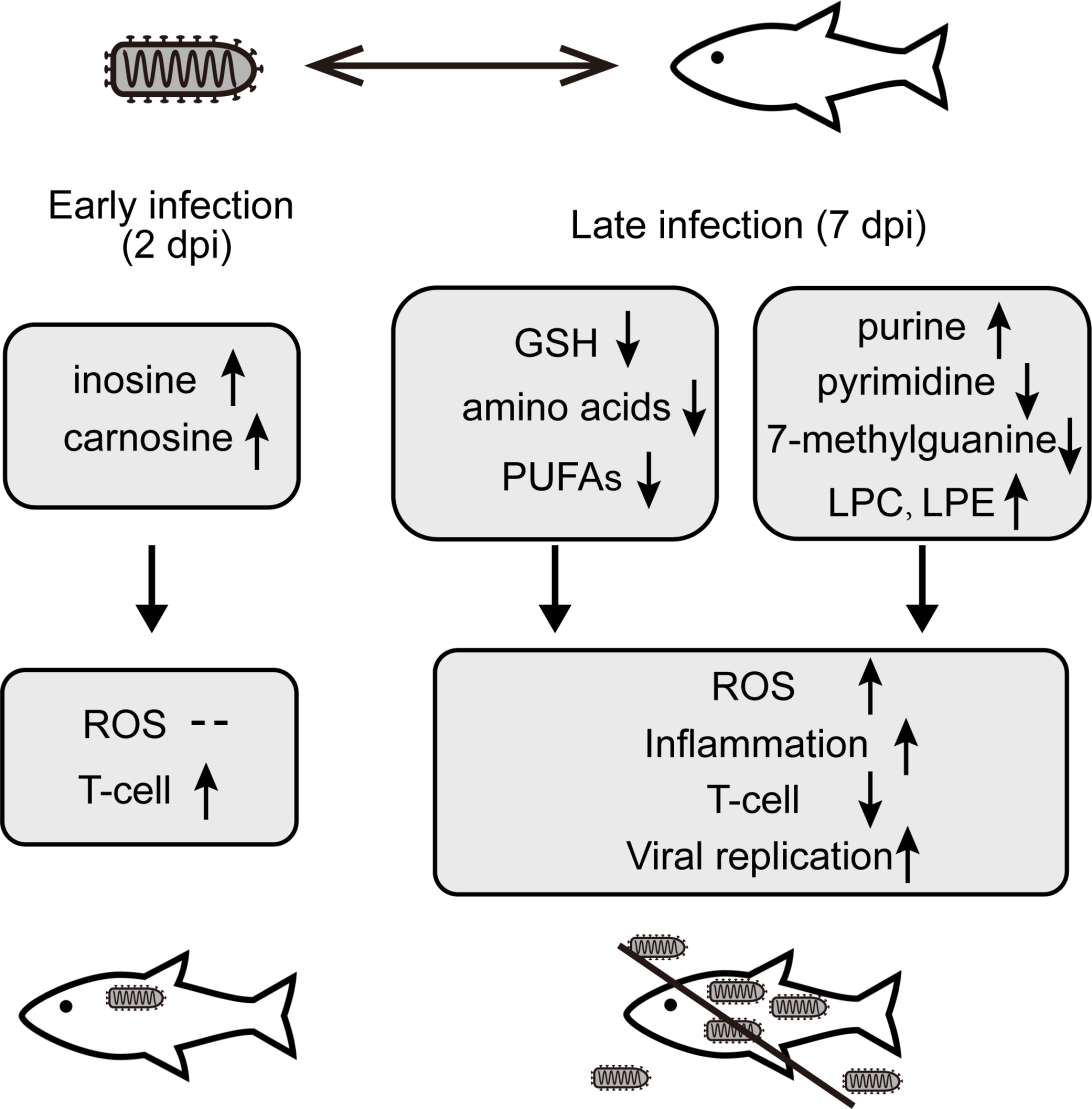
The changes of key metabolites and corresponding mechanism involved in the interaction between the olive flounder and HIRRV at different stages of infection.

At the late stage of viral infection, nucleotide metabolism pathways were also disturbed. Purine and pyrimidine exhibited two different patterns subjected to HIRRV infection. Majority of metabolites involved in purine pathway, especially guanine, hypoxanthine, and deoxyinosine were up-regulated. While, cytidine and dCMP, related to pyrimidine metabolism, were down-regulated (**Figure 4**). The different patterns of purine and pyrimidine might lead to imbalance of nucleotide pool (**Figure 7**). The ratio of purine and pyrimidine usually strictly controlled, therefore viral infection might induce the imbalance of purine and pyrimidine, and further contribute to the cell death (39).

After viral infection, methylation of guanine was observed. We observed a 13-fold decrease in 7-methylguanine, despite an increased guanine level (**Figure 4** and **Figure S2**). The presence of the 5’-cap structure of mRNA, which is formed by 7-methylguanine, is crucial for an effective immune response. The composition of 5’ end RNA, including the 5’-cap and its methylation status, has been recognized as a crucial determinant in host recognition (40). It is widely documented that viruses have evolved mechanisms to modify the N7 position of the capped guanine, enabling viral RNAs to evade detection by the host’s innate immune system (41). Therefore, the observed decline in 7-methylguanine levels may be due to HIRRV’s increased demand for cap structure formation, allowing it to evade host immune responses.

Meanwhile, 7-methylguanine is also an inhibitor of DNA repair enzyme, poly (ADP-ribose) polymerase (PARP) (42). Previous studies have shown that PARPs could promote the replication of a variety of RNA viruses, including HIV, influenza A virus, porcine reproductive and respiratory syndrome virus, Sindbis virus and T-lymphotropic virus (43). Hence, the prominent reduction of 7-methylguanine might attenuate the inhibition of PARPs, which further enhance the proliferation of HIRRV and lead to cell death (**Figure 5A**).

Our findings suggest that 7-methylguanine plays a critical role in HIRRV infection, either by assisting the virus in evading the host’s immune system or promoting viral RNA replication through inhibition of PARP. In our exogenous addition assays, adding 7-methylguanine also induced the burst of HIRRV particles. Therefore, targeting 7-methylguanine may be a promising therapeutic strategy for treating HIRRV infection.

### 4.4 Antioxidant changed along with viral infection

ROS are free radicals and are considered natural byproducts of various biological activities. The pathogenic infection could tip the balance between the ROS and antioxidants (44). Our metabolomic analyses revealed several important metabolites involved in ROS.

Two days after HIRRV infection, the up-regulation of inosine and carnosine was observed, both of which are considered as natural antioxidant (**Figure S2**) (45, 46). The increased antioxidants of olive flounder at the early stage of viral infection suggest that antioxidants might be used to neutralize elevated ROS induced by HIRRV. Along with viral infection, the concentration of HIRRV was considerably boosted 7 days post-infection (**Figure 1**) and the cumulative mortality rate of the flounder reached up to 20% (5). It has been reported that numerous retroviruses, DNA viruses, and RNA viruses can induce oxidative stress in infected cells and eventually lead to cell death (47, 48). At the metabolic level, several metabolites involved in ROS reduced including signature antioxidants GSH. Both our RT-qPCR result and previous transcriptomic analysis demonstrated an up-regulation of genes involved in GSH utilization, such as GST and GGT1_5 (Glutathione hydrolase 1 proenzyme) (**Figure S3**) (8). The reduction of GSH implies an increasing demand for antioxidants due to the accumulated ROS level (49). In agreement with our finding, a previous study also reported the suppression of the glutathione pathway and an increase in ROS production of the olive found in response to VHSV infection (16). The complex interaction and potential molecular mechanisms among viruses and the cellular redox response of aquaculture species deserve further investigation.

### 4.5 Global changes of lipid profile

In our analysis, lipids are remarkably affected by viral infection at the late stage, occupying the largest proportion of disturbed metabolites. The composition of lipids could modulate host receptor binding and affect viral replication (50). Viral infection could disrupt membrane integrity during entry or lysis, and influence the composition of lipidome (51).

Significant upregulation of different types of LPE and LPC along with viral infection were found (**Figure 4**). Meanwhile, an substantial increase in LPC after within HIRRV infection was unfolded by ELISA at cellular level, indicating that the pathogen invasion caused a significant elevation of LPC (**Figure 6C**). Many studies have revealed that viral infection can enrich LPE and LPC, and further stimulate virus replication, indicating key roles of LPE and LPC in viral infection (52). For example, extremely high levels of LPE and LPC were found during SARS-CoV-2 infection (53). Similarly, the infection of coxsackievirus A16 (CV-A16) could also up-regulate LPE (54). The significant increase in lipid metabolites implied that at the late stage of viral infection HIRRV hijacked host cells for its reproduction, and the phospholipid may be an important component for the massive replication of the pathogen in late stages (**Figure S2**). In addition, we also found that all of the up-regulated LPC were unsaturated, with at least four degrees of unsaturation. LPC can be recycled to PC, the most abundant phospholipid of the cell membrane (55). Therefore, the elevation of unsaturation of LPC could increase the fluidity of the membrane and decrease the stability of the host cell membrane, which might be beneficial for HIRRV to lyse the flounder cell membrane.

Various polyunsaturated fatty acids (PUFAs) also declined in response to viral infection (**Figure S2**). The syntheses of both omega-3 and omega-6 types of PUFAs were inhibited, including α-linolenic acid, linoleic acid, stearidonic acid, docosahexaenoic acid (DHA), eicosapentaenoic acid (EPA), γ-linolenic acid, arachidonic acid (ARA) (**Figure 4** and **Figure S2**). PUFAs, especially omega-3 type, have been known to reduce inflammation during chronic disease (56). The EPA and DHA can attenuate the activation of NF-κB pro-inflammatory transcription factors (57). The overall decreased PUFAs imply the amplified inflammatory reaction in olive flounder at the late stage of viral infection, which may be one of the reasons for cell death (58).

Together, HIRRV infection changes the global lipid profile of host cells. However, the role of lipids in viral infection is complex. Additional studies in fish of economic value are essential to elucidate the role of lipids in regulating the immune response to viral infection.

## 5 Conclusion

In this study, UPLC-MS-based metabolomic approach was applied to investigate the metabolic changes of olive flounder in response to HIRRV infection. Distinct metabolic profiles were displayed in the serum of olive flounder along with viral infection (**Figure 7**). Addition of inosine and carnosine could be a potential strategy to prevent the proliferation of HIRRV in the early stage. In particular, the deduction of amino acids helps HIRRV to proliferation and impede the host immune system. The modification and imbalance of nucleotide, especially the reduction of 7-methylguanine, and up-regulation of LPC and LPE may facilitate HIRRV infection and replication, contributing to the mortality of the fish. Significant alternations in metabolites included key amino acids, GSH, which were involved in the immune and antioxidant systems of flounder. This is the first time that a metabolomic approach was used to investigate the mutual antagonism between HIRRV and flounder, providing a new perspective on the pathogenesis of HIRRV infection in the flounder. Significant changes of specific metabolites hijacked by HIRRV may provide a basis for the diagnosis of the disease and a new strategic direction for the development of anti-viral therapy against HIRRV infection.

## Data availability statement

The original contributions presented in the study are included in the article/**Supplementary Material**, further inquiries can be directed to the corresponding author.

## Ethics statement

The animal study was approved by the Instructional Animal Care and Use Committee of the Ocean University of China (Permit Number: 20180101). The study was conducted in accordance with the local legislation and institutional requirements.

## Author contributions

YZ and BG designed the study. BG, FP, HW, ZZ and JS performed the experiments. BG wrote the manuscript. JX, XT, and YZ revised the manuscript. All authors discussed the results and contributed to the final manuscript. All authors contributed to the article and approved the submitted version.

## Supplementary material

Supplementary Figure 1 The symptom of the diseased *Paralichthys Olivaceus*. **(A)** Observations revealed the presence of body surface blackening, fin ray hemorrhage, and body surface ulceration. **(B)** Dissection revealed vasodilation and hemorrhage of the visceral organs, accompanied by the production of ascites.

Supplementary Figure 2 The abundance of select metabolites of the olive flounder along with HIRRV infection. Each dot represents an individual sample (*: *p* < 0.05, **: *p* < 0.01, ***: *p* < 0.001, ****: *p* < 0.0001).

Supplementary Figure 3 Schematic representation of the relationship between amino acids and glutathione. **(A)** Metabolites on red background were significantly upregulated and those on blue background were significantly downregulated. Enzymes are shown in bold red. **(B)** Changes in specific metabolites and genes are shown in small Figures (*: *p* < 0.05, **: *p* < 0.01, ***: *p* < 0.001, ****: *p* < 0.0001).

Supplementary Figure 4 The cytotoxicity of the addition of exogenous metabolites to cells (percentage of cell viability ± SD, *: *p* < 0.05). The cell survival rate of all the experimental groups with exogenous metabolites was above 80%, indicating neglectable toxicity of these metabolites.
