# Supplementary figures and images for: Untargeted LC-MS metabolomics reveals the metabolic responses in olive flounder subjected to hirame rhabdovirus infection
Source: Front Immunol. 2023 Aug 28;14:1148740. doi: 10.3389/fimmu.2023.1148740 (PMC10498126; doi:10.3389/fimmu.2023.1148740)

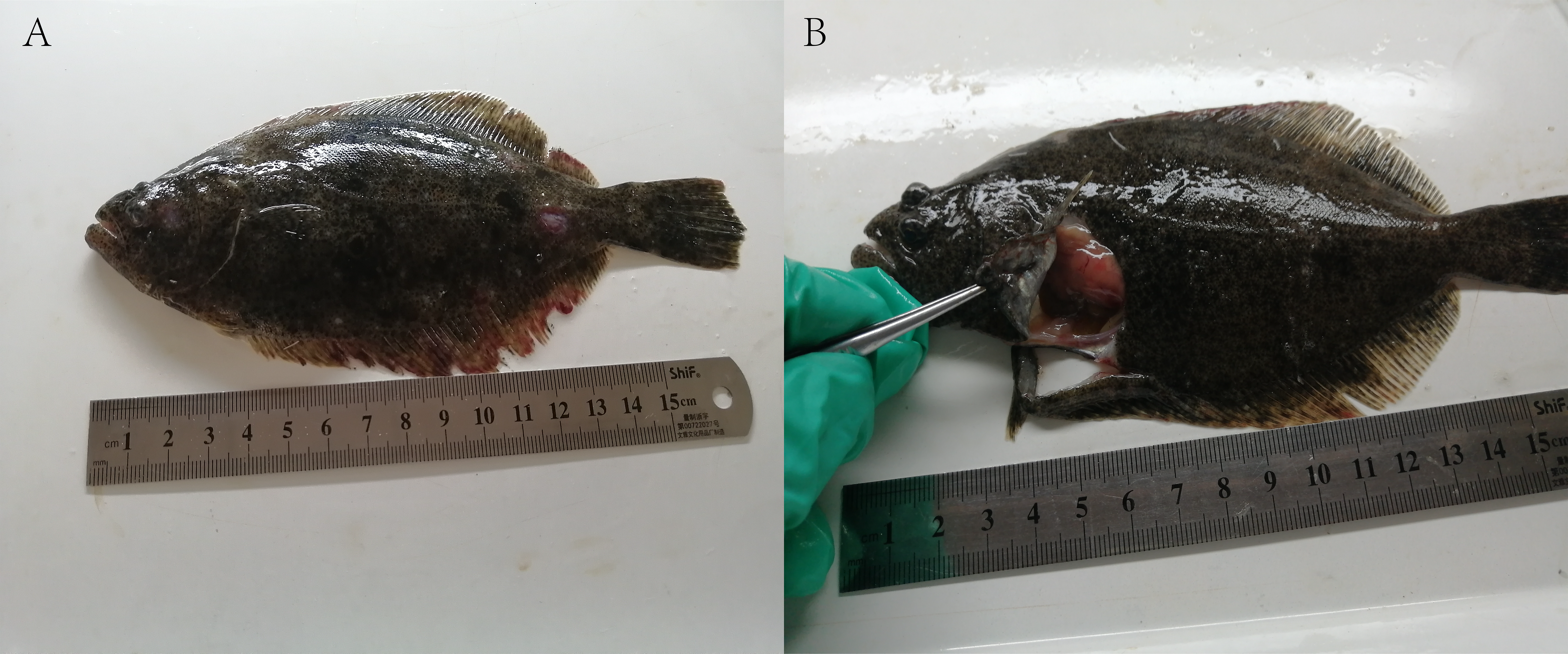

Supplement: Supplementary Figure 1 — The symptom of the diseased Paralichthys Olivaceus. (A) Observations revealed the presence of body surface blackening, fin ray hemorrhage, and body surface ulceration. (B) Dissection revealed vasodilation and hemorrhage of the visceral organs, accompanied by the production of ascites. [file Image_1.png]

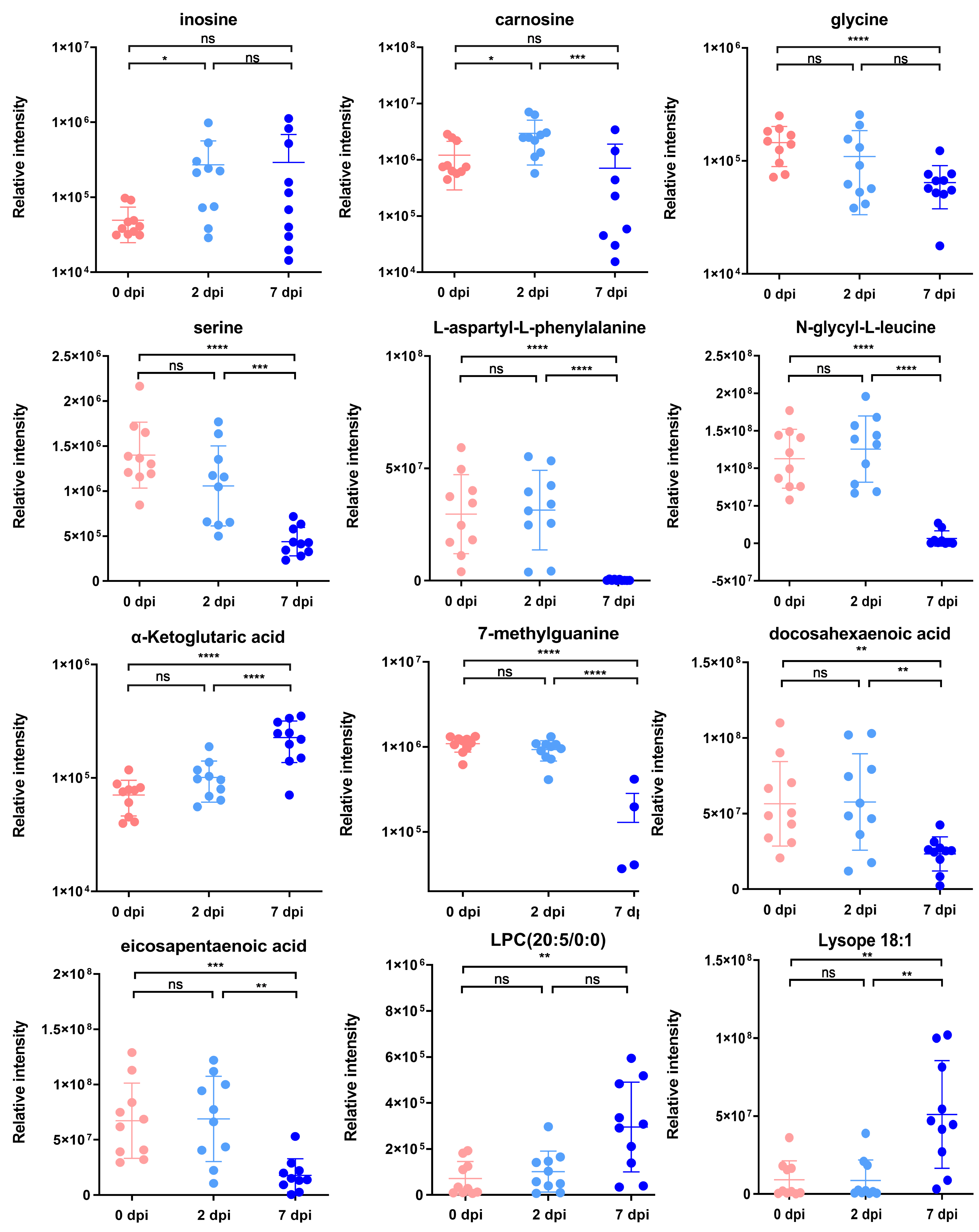

Supplement: Supplementary Figure 2 — The abundance of select metabolites of the olive flounder along with HIRRV infection. Each dot represents an individual sample (*: p < 0.05, **: p < 0.01, ***: p < 0.001, ****: p < 0.0001). [file Image_2.png]

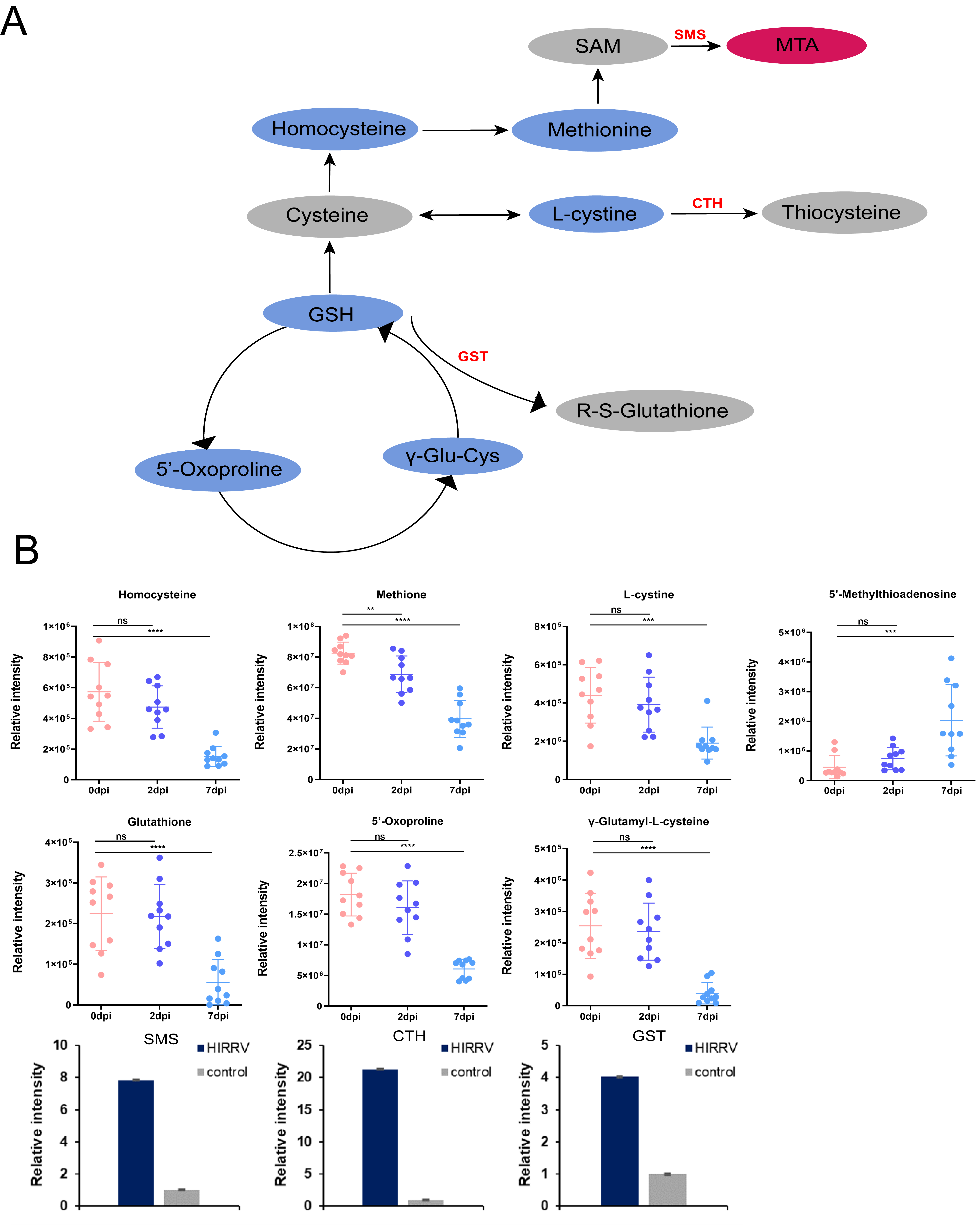

Supplement: Supplementary Figure 3 — Schematic representation of the relationship between amino acids and glutathione. (A) Metabolites on red background were significantly upregulated and those on blue background were significantly downregulated. Enzymes are shown in bold red. (B) Changes in specific metabolites and genes are shown in small Figures (*: p < 0.05, **: p < 0.01, ***: p < 0.001, ****: p < 0.0001). [file Image_3.png]

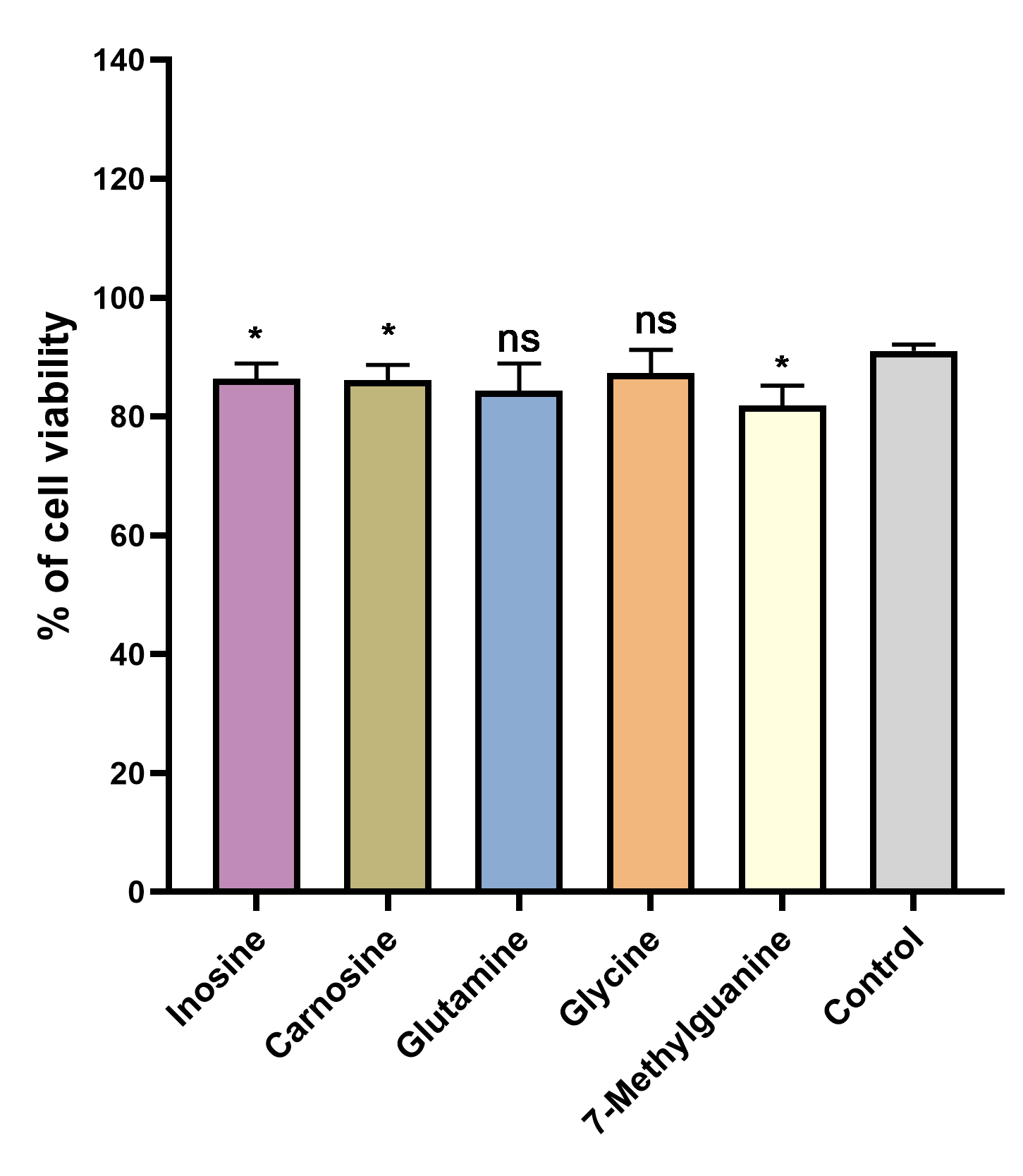

Supplement: Supplementary Figure 4 — The cytotoxicity of the addition of exogenous metabolites to cells (percentage of cell viability ± SD, *: p < 0.05). The cell survival rate of all the experimental groups with exogenous metabolites was above 80%, indicating neglectable toxicity of these metabolites. [file Image_4.tif]
